# Heterogeneity of resting and hyperemic myocardial blood flow in healthy volunteers: a quantitative CMR perfusion pixel-map study

**DOI:** 10.1186/1532-429X-16-S1-P21

**Published:** 2014-01-16

**Authors:** Anders M Greve, Li-Yueh Hsu, Sujethra Vasu, W Patricia Bandettini, Andrew E Arai

**Affiliations:** 1 Department of Health and Human Services, Advanced Cardiovascular Imaging Laboratory, National Heart, Lung, and Blood Institute, National Institutes of Health, Bethesda, Maryland, USA; 2 Section of Cardiology, Wake Forest University School of Medicine, Winston-Salem, North Carolina, USA

## Background

An accurate description of the heterogeneity in myocardial blood flow (MBF) by CMR is needed for understanding the physiology of perfusion variability.

## Methods

Quantitative CMR perfusion was performed at 1.5T in 17 healthy volunteers under baseline (rest) and adenosine hyperemia (stress). Median filters with different kernel size were used to estimate MBF at different resolutions (0.07 g, 0.27 g, 0.61 g, 1.1 g of myocardium). MBF heterogeneity was evaluated as the relative dispersion ([RD] = standard deviation/mean) on basal- and mid-ventricular slices for each subject. Paired t-tests and linear mixed-models were used to account for within-subject effects.

## Results

**Figure 1:**
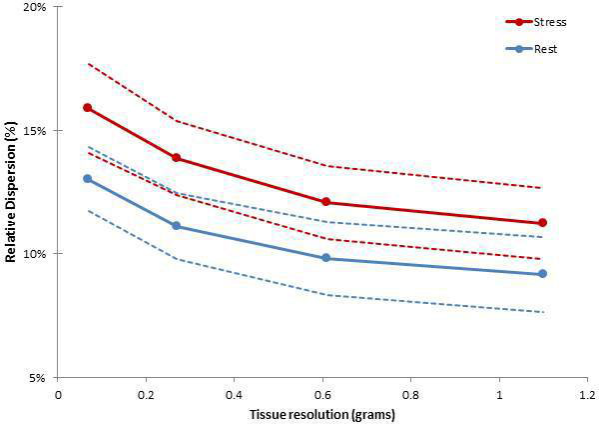
**Heterogeneity of myocardial blood flow varies as a function of tissue analysis resolution (p < 0.001 for each)**. Relative dispersion of myocardial flow (Y-axis) at different tissue analysis resolutions (X-axis) and under baseline (rest) and adenosine hyperemia (stress). Circle indicates mean and dotted lines the upper and lower 95% confidence limits for each tissue resolution.

All normal volunteers had Framingham scores <1%. MBF at rest was 1.1 ml/g/min (95% CI: 0.9 to 1.2 ml/g/min) vs. adenosine stress 2.8 ml/g/min (95% CI: 2.4 to 3.1 ml/g/min [p < 0.001]). At the intrinsic image acquisition resolution (1 voxel = 0.07 g), the RD was 13.0% (95% CI: 11.7 to 14.3%) at rest vs. 15.9% (95% CI: 14.1 to 17.7%) at adenosine stress (p = 0.004). For increasing voxel sizes, the RD of MBF under rest and stress conditions decreased in a highly-significant pattern (Figure 1). There were no detectable differences in pairwise comparisons of RD between basal and mid-slices at rest or under hyperemic conditions (all p = NS).

## Conclusions

Healthy myocardium displays resolution-dependent hetereogeneity of MBF at rest that increases during hyperemia. MBF heterogeneity by quantitative CMR is lower than that reported by microsphere data for equal tissue weight. Furthermore, this analysis is, to the best of our knowledge, 4 times higher resolution than any microsphere or PET analysis.

## Funding

This project was funded by the Intramural research program of the National Heart Lung and Blood Institute at the National Institutes of Health.

